# Molecular cloning, structural and expression profiling of *DlRan* genes during somatic embryogenesis in *Dimocarpus longan* Lour.

**DOI:** 10.1186/s40064-016-1887-0

**Published:** 2016-02-25

**Authors:** Zhizhen Fang, Chengchun Lai, Yaling Zhang, Zhongxiong Lai

**Affiliations:** Institute of Horticultural Biotechnology, Fujian Agriculture and Forestry University, 15 Shangxiadian Road, Cangshan District, Fuzhou, 350002 Fujian China

**Keywords:** Cloning, *Dimocarpus longan*, Gene expression, Ras-related nuclear protein, Somatic embryogenesis

## Abstract

To clone and examine expression profiles of *DlRan* genes during somatic embryogenesis in *Dimocarpus longan* Lour. Thirty cDNA sequences and two genomic sequences encoding DlRan proteins were isolated from longan embryogenic cultures. Structural analysis of *DlRan* genes revealed that the longan *Ran* gene family is more expanded than that of Arabidopsis. Expression analysis of *DlRan* genes during somatic embryogenesis uncovered a high abundance of *DlRan* genes in early embryogenic cultures and heart- and torpedo-shaped embryos. The expression of *DlRan* genes in embryogenic calli was affected by exogenous 2,4-dichlorophenoxyacetic acid treatment. *DlRan* is involved in 2,4-D induced somatic embryogenesis and development of somatic embryos in longan.

**Electronic supplementary material:**

The online version of this article (doi:10.1186/s40064-016-1887-0) contains supplementary material, which is available to authorized users.

## Background

Ras-related nuclear protein (Ran) is a highly conserved, small GTPase family that is essential to multiple cellular processes in eukaryotes (Clarke and Zhang 2008). The roles of Ran have been extensively researched and well documented in animals. In contrast, little is known about Ran in plants.

Plant Ran proteins share high homology and perform similar functions in the regulation of mitotic progress with their counterparts in yeast and animals (Lü et al. 2011; Lee et al. 2008; Wang et al. 2006; Zang et al. 2010). Furthermore, Ran is involved in mediating responses to external stimuli, such as heat, salt and drought stresses (Ferreira et al. 2006; Jiang et al. 2007; Xu and Huang 2008, 2010; Yoshimura et al. 2008; Zang et al. 2010). Inhibition expression of *OsRan2* in rice leads to pleiotropic developmental abnormalities (Chen et al. 2011; Zang et al. 2010). These results suggest that Ran is crucial to plant growth and development.

Longan (*Dimocarpus longan* Lour.), an evergreen fruit tree of great commercial value, is distributed in subtropical and tropical countries (Matsumoto 2006; Zheng et al. 2009). Longan embryo development is of great scientific interest because of its role in fruit quality and yield. The developmental regulation of Ran during the middle stage of longan somatic embryogenesis (SE) implies a role for Ran in this process (Fang et al. 2011). Furthermore, *Ran* has been proposed as a target for breeding and production improvement in longan (Fang et al. 2014) because of its role in delaying flowering and enhancing cold tolerance in other plants (Chen et al. 2011; Wang et al. 2006). Nevertheless, cloning and characterization of longan *Ran* has not yet been reported.

In this study, 30 cDNA sequences and two genomic sequences encoding DlRan proteins were isolated. We analyzed the structures of *DlRan* genes, and investigated their expression profiles during SE and under exogenous 2,4-dichlorophenoxyacetic acid (2,4-D) treatment. On the basis of our results, we propose that DlRan is involved in cell division during longan SE and participates in 2,4-D-induced SE through signal transduction.

## Methods

### Plant materials

The establishment and maintenance of our longan embryogenic callus line “Honghezi” was described in Lai et al. (2000). The synchronization of embryogenic cultures at different developmental stages was carried out as described previously (Fang et al. 2014). All cultures were kept in dark conditions at 25 ± 1 °C.

### RNA extraction

Total RNA was extracted from embryogenic cultures using TriPure Isolation Reagent (Roche Molecular Biochemicals, Basel, Switzerland) and then treated with DNase I (Takara, China) to remove genomic DNA.

### 5′ and 3′ rapid amplification of cDNA ends (RACE)

A 469-bp cDNA fragment of *DlRan* (*Ran* fragment 1) was obtained by reverse-transcription PCR with degenerate primers (RanF1 and RanR1) generated according to mass spectrographic analysis results in our previous study (Fang et al. 2011). 5′ and 3′ RACE were performed to generate full-length gene transcripts. The 3′ RACE was performed using a First-Strand cDNA synthesis kit (Fermentas). 12 3′-ends of *DlRan* cDNAs were obtained using specific primers designed from *Ran* fragment 1 (Table 1). Multiple alignment of these 3′ ends indicated the existence of *DlRan* homologs. A specific primer, RanR2, was designed according to the isolated 3′ ends, and a new *DlRan* fragment (*DlRan* fragment 2) was obtained using RanF1 and RanR2. Primers RanF8 and RanF9 were generated according to *DlRan* fragments 1 and 2 and used for 3′ RACE, yielding three additional *DlRan* cDNA 3′ ends (Table 1). A 5′ RACE was performed using a GeneRacer kit (Invitrogen). Specific primers were designed according to the isolated *DlRan* fragments and 3′-RACE products of *DlRan* and used for 5′ RACE. Primers and corresponding 5′-RACE products are indicated in Table 1. For amplification of full-length *DlRan* cDNAs, gene-specific primers were generated according to the *DlRan* 5′ and 3′ ends, with cDNAs synthesized from the GeneRacer kit used as templates. Specific primers used are listed in Table 2 and Additional file 1: Figure S1.

**Table 1:** Specific primers used for 3′ and 5′ RACE and corresponding products

Specific primers	Products
Outer primer: RanF2 Nested primer: RanF3	Ran3′-1, Ran3′-2
Outer primer: RanF4 Nested primer: RanF5	Ran3′-3, Ran3′-4, Ran3′-5, Ran3′-6, Ran3′-7, Ran3′-8, Ran3′-9, Ran3′-10, Ran3′-11, Ran3′-12
Outer primer: RanF8 Nested primer: RanF9	Ran3′-13, Ran3′-14, Ran3′-15
Outer primer: RanR3 Nested primer: RanR4	Ran5′-1, Ran5′-2, Ran5′-3, Ran5′-4, Ran5′-5
Outer primer: RanR5 Nested primer: RanR6	Ran5′-6, Ran5′-7, Ran5′-8, Ran5′-9, Ran5′-10, Ran5′-11
Outer primer: RanR7 Nested primer: RanR8	Ran5′-12
Outer primer: RanR9 Nested primer: RanR10	Ran5′-13, Ran5′-14, Ran5′-15
Outer primer: RanR12 Nested primer: RanR13	Ran5′-16, Ran5′-17
Outer primer: RanR11 Nested primer: RanR13	Ran5′-18

**Table 2:** Primers used in this study

Name	Primer sequences (5′–3′)	Name	Primer sequences (5′–3′)
RanF1	GTNGGNGAYGGNGGNACNGG	RanR7	CACCAGAGGAGCACAAAAAGCAGCAT
RanF2	CGTTTCTACTGCTGGGATAC	RanR8	CTGCAACTGTTCTCTATTCAAATGTGT
RanF3	CTGCCAAGAGCAACTACAAT	RanR9	TGTTCATCAACCCCAACTCCAACAAT
RanF4	CAGGAGAAGTTTGGTGGTCT	RanR10	CAATCACACAATTCCCCATCCTGCT
RanF5	GATGTTACTGCTCGCTTGAC	RanR11	AACNTGCTTNGCNTTCACTTGCCT
RanF6	CTCTGCGGAAACAARGTTGATGT	RanR12	CANACCCNGCAAAGATNACNGTG
RanF7	GAARCCTTTCTTGTACCTTGCC	RanR13	TGATNATNGCACATTGCCCATGGAT
RanF8	CTCTGCGGAAACAARGTTGATGT	RanR14	TTTATGAGGCAACACTGGTTCAG
RanF9	GAARCCTTTCTTGTACCTTGCC	RanR15	CCCCCTTTTTTTCCATGCAAATT
RanF10	CAAGACCAAAAGCTCTCCCTCTAAT	RanR16	CCCCCCCTTTTTTTTTTAGGAG
RanF11	CGCTCTCAGAACCAAACCAAGAAG	RanR17	CCCCTTTTTTTACGGAGCAAC
RanF12	GGTGCTTATTGATACATTTCTCC	RanR18	CCCCTTGAAA ACCAGATAAA ATG
RanF13	CACTCTAATTGCCTTCCTACTTCGT	RanR19	CCCCCTTTTTTTTGGTATGTAAG
RanF14	GGCAGCAGAGAGAGAGAATC	RanR20	CCCCCCTTTTTTTTAACAAGACC
RanF15	GATTGGCTGTTGTTTTGAAGAAG	RanR21	CCCCTTTTTTTATCCTCAACACC
RanF18	CAAGACCAAAAGCTCTCCCTCT	RanR22	CCCCCCCTTTTTTCAGATAATAT
RanR1	GRTCNCCNGCNAGYTTNCGNGC	RanR23	CCCCCTTTTTATACTCAACTATC
RanR2	GCATCATCATCGTCATCTGG	RanR24	TCCCCATCCTGCTGTTTTACTCGA
RanR3	CCTGTGGAATGTAACCTGCT	RanR25	CCCCCCTTTTTTTTTTTTTTTAGGAGAA
RanR4	CCTTCACTTGCCTATTCCTC	RanR26	CGGAGCAACGCTTAAAACATCCTACA
RanR5	GTCAAGCGAGCAGTAACATC	RanR29	CAGCGTAGGGGGAGCCGAATGAAT
RanR6	AGACCACCAAACTTCTCCTG	RanR30	CCAGCCTGCAACTGTTCTCTATTCA
5P	CGACTGGAGCACGAGGACACTGA	AUAP	GCCACGCGTCGACTAGTAC
5NP	GGACACTGACATGGACTGAAGGAGTA		

### DNA extraction and isolation of genomic DNA encoding DlRan

Total genomic DNA was isolated from longan embryogenic calli with a Plant Genomic DNA kit (Tiangen, China). A 2389-bp *DlRan* DNA sequence was obtained using specific primers (RanF18 and RanR29; Table 2) and Takara LA *Taq* (Takara) and was designated as *DlRan3A* (GenBank accession no. JQ775539). The genomic sequence of *DlRan3B* (JQ279697) has been characterized previously (Fang et al. 2013).

### Quantitative real-time PCR analysis

cDNAs were synthesized with random primers and Oligo dT Primer using a SYBR ExScript kit (Takara). Real-time PCR amplifications were performed on a Lightcycler 480 system (Roche Applied Science, Switzerland) in 20-µl total volumes containing 10 µl of 2× SYBR Premix Ex *Taq* II (Takara), 1 µl cDNA (1:10 dilution), and 0.4 µl of each 0.20-µM primer. PCR conditions were as follows: denaturation at 95 °C for 30 s, followed by 40 cycles of 95 °C for 5 s, 60 °C for 30 s and 72 °C for 30 s. Reactions were run in triplicate. *EF*-*1a* and *Fe*-*SOD*, the most stable genes selected by Lin and Lai (2010), were used as endogenous controls. Expression data were analyzed with geNORM (version 3.5) (Vandesompele et al. 2002). The high sequence similarity among isolated *DlRan* transcripts made it very difficult to design specific primers to detect their expression. We found that the identified *DlRan* transcripts could be divided into two types, N (asparagine) and D (aspartic acid), based on the tenth residue in their predicted amino acid sequences. Specific primers based on the 5′-end proximal region of these N and D *DlRan* transcript sequences (Additional file 2: Figure S2) were designed and used for qRT-PCR analyses. Primer pairs used for qRT-PCR analyses are listed in Table 3.

**Table 3:** Primers used for qRT-PCR analysis

Specific primer	Primer sequences (5′–3′)
N type *DlRans*	Forward: AAGGACAGCTCTCATGGCTTTGC
	Reverse: TGCCTCCATCACCGACGATGAC
D type *DlRans*	Forward: TAGTGATCGTCGGCGATGGTGG
	Reverse: TGCAGTGTCCCAGCAATAGAAGCG
*Fe*-*SOD*	Forward: GGTCAGATGGTGAAGCCGTAGAG
	Reverse: GTCTATGCCACCGATACAACAAACCC
*EF*-*1a*	Forward: GATGATTCCCACCAAGCCCAT
	Reverse: GGGTCCTTCTTCTCAACACTCT

### Treatment of embryogenic calli with 2,4-D

Embryogenic calli cultured on M0 medium (Murashige-Skoog basal salts, 2% sucrose and 6 g/L agar, pH 5.8) supplemented with 1 mg 2,4-D/l were transferred and maintained for 24 h on M0 medium or M0 medium supplemented with either 0.5, 1.5 or 2.0 mg/l of 2,4-D. All samples were frozen in liquid nitrogen after harvesting and stored at −80 °C.

### Bioinformatics analysis

Predicted protein sequences were analyzed and theoretical isoelectric points (pIs) and mass values of mature peptides were calculated using the PeptideMass program (http://us.expasy.org/tools/peptidemass.html). Amino acid sequence alignment was performed using DNAMAN software. A phylogenetic tree of Ran proteins was constructed using MEGA5 software.

## Results

### Cloning of *DlRan* cDNAs from torpedo-shaped somatic embryos of longan

Fifteen 3′ ends of *DlRan* genes were obtained through 3′ RACE. Alignment of these 3′ ends indicated the existence of sequence polymorphism in *DlRan* gene open reading frames (ORFs) and 3′ untranslated regions (UTRs) (Additional file 3: Figure S3). 18 5′ ends of *DlRan* genes were obtained using RNA ligase-mediated RACE (Additional file 4: Figure S4). Using primers designed from the isolated 5′ and 3′ ends, we isolated 30 *DlRan* transcripts from torpedo-shaped somatic embryos in longan and deposited their sequences in GenBank (Table 4).

**Table 4:** GenBank accession numbers of *Ran* cDNAs and primer pairs used for their amplifications

Name	Accession no.	Primer pairs (forward/reverse)
*DlRan3A*-*1*	JF461272	RanF10/RanR14
*DlRan3A*-*2*	JF461273	RanF10/RanR15
*DlRan3A*-*3*	JF461274	RanF10/RanR16
*DlRan3A*-*4*	JF461275	RanF10/RanR17
*DlRan3A*-*5*	JF461276	RanF10/RanR18
*DlRan3A*-*6*	JF461277	RanF10/RanR19
*DlRan 3A*-*7*	JF461278	First PCR: RanF10/3P Nested PCR: RanF11/3NP
*DlRan3A*-*8*	JF461279	First PCR: RanF10/3P Nested PCR: RanF11/3NP
*DlRan3A*-*9*	JF461280	First PCR: RanF10/3P Nested PCR: RanF11/3NP
*DlRan A*-*10*	JF461281	First PCR: RanF10/3P Nested PCR: RanF11/3NP
*DlRan3A*-*11*	JF461282	First PCR: RanF10/3P Nested PCR: RanF11/3NP
*DlRan3A*-*12*	JQ861699	First PCR: 5P/RanR25 Nested PCR: 5NP/RanR26
*DlRan3A*-*13*	JQ775533	RanF12/RanR24
*DlRan3A*-*14*	JQ775532	RanF12/RanR24
*DlRAN3B*-*1*	HM773390	RanF18/RanR20
*DlRan3B*-*2*	JF461283	RanF18/RanR21
*DlRan3B*-*3*	JF461284	RanF18/RanR14
*DlRan3B*-*5*	JF461286	RanF13/RanR21
*DlRan3B*-*6*	JF461287	RanF13/RanR22
*DlRan3B*-*7*	JF461288	RanF13/RanR14
*DlRan3B*-*8*	JQ775530	RanF14/RanR30
*DlRan3B*-*9*	JQ775531	RanF14/RanR30
*DlRan3C*-*1*	JF461289	RanF13/RanR23
*DlRan3C*-*2*	JF461290	RanF13/RanR23
*DlRan3C*-*3*	JF461291	RanF13/RanR23
*DlRan3D*-*1*	JF461292	RanF13/RanR19
*DlRan3D*-*2*	JF461293	RanF13/RanR17
*DlRan3E-1*	JF461294	RanF10/RanR20
*DlRan3F*-*1*	JQ775527	RanF10/RanR20
*DlRan3G*-*1*	JQ775528	RanF10/RanR20

### Sequence analyses and molecular characterization of *DlRan* genes

**Fig. 1:**
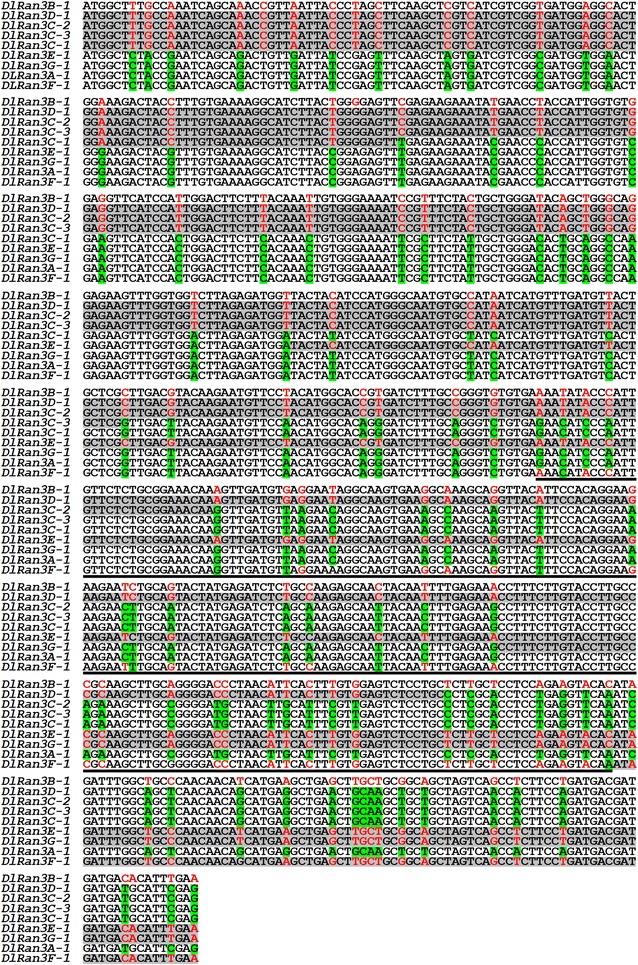
Multiple alignments of the open reading frame sequence of *DlRan* genes. Sequence fragments consistent with *DlRan3B*-*1* were indicated with *grey shadow*, sequence fragment of *DlRan3F*-*1* that is not consistent with *DlRan3B*-*1* nor *DlRan3A*-*1* were highlighted with *underline*, different bases among the aligned sequences are indicated by *colors*

Sequence analysis indicated that all of the isolated *DlRan* transcripts contained a 663-bp ORF. The 3′ UTRs of the isolated *DlRan* transcripts lack the typical AATAAA polyadenylation signal. The isolated *DlRan* cDNAs were divided into nine groups according to their ORF sequences (Fig. 1). *DlRan3A*s, *DlRan3B*s, *DlRan3C*-*1*, *DlRan3C*-*2*, *DlRan3C*-*3*, *DlRan3D*s, *DlRan3E*-*1*, *DlRan3F*-*1* and *DlRan3G*-*1* had unique ORFs (Fig. 1). Sequence alignment showed that the first half of sequences of *DlRan3D*-*1*, *DlRan3C*-*1*, *DlRan3C*-*2* and *DlRan3C*-*3* were identical to that of *DlRan3B*-*1*, while the second half of sequences of these cDNAs were identical to that of *DlRan3A*-*1*. In contrast, the first half of *DlRan3E*-*1* and *DlRan3G*-*1* sequences were identical to *DlRan3A*-*1*, and the second half of sequences of these cDNAs were identical to that of *DlRan3B*-*1*. One fragment of *DlRan3F*-*1* was identical to neither *DlRan3A*-*1* nor *DlRan3B*-*1* (Fig. 1). These results prompted us to explore whether the transcripts identified in the present study were alternative spliced isoforms produced by the same gene or were instead transcribed from different genes.

**Fig. 2:**
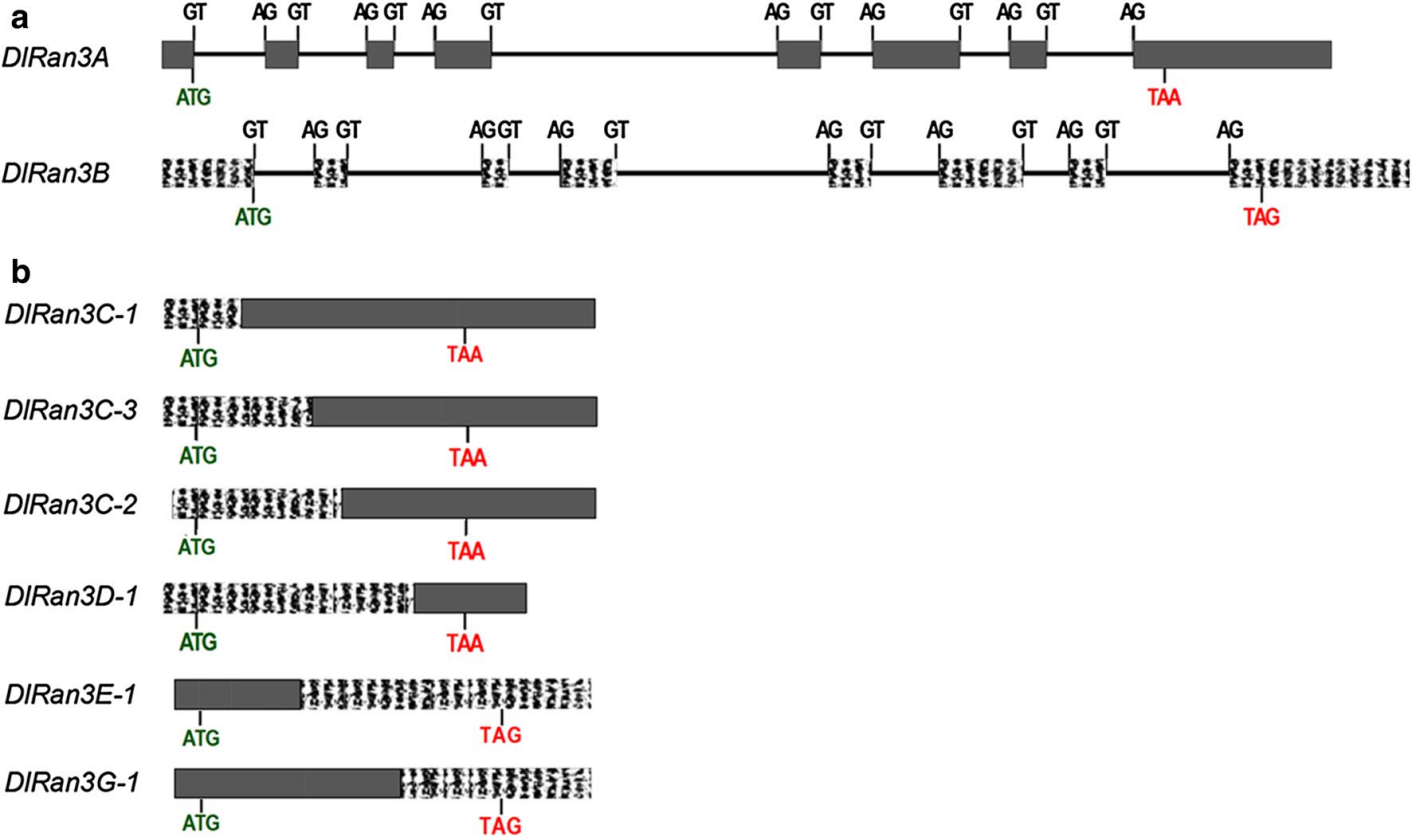
Alignments of *DlRan* cDNAs and genomic DNA sequences. **a** Exon–intron organization of *DlRan3A* and *DlRan3B*. *Bold lines* represent introns, *grey* and *texture boxes* indicate exons, GTs and AGs represent bases close to the identical sequences, start and termination codons were indicated in *green* and *red* character respectively. **b** Schematics of alignments between *DlRan* cDNAs and genomic DNA sequences

To determine exon and intron organization of *DlRan* cDNAs, we try to isolate genomic sequences of *DlRan* genes and only 2 *DlRan* sequences (*DlRan3A* and *DlRan3B*) were obtained. The comparative analysis of *DlRan* genomic and cDNA sequences indicated that *DlRan3A*-1–*DlRan3A*-14 was derived from *DlRan3A* and that *DlRan3B*-1–*DlRan3B*-3 and *DlRan3B*-5–*DlRan3B*-9 were derived from *DlRan3B*. As indicated in Fig. 2, both *DlRan3A* and *DlRan3B* contained 8 exons. Interestingly, the first half of the sequences of *DlRan3D*-*1*, *DlRan3C*-*1*, *DlRan3C*-*2* and *DlRan3C*-*3* were identical to the genomic sequence of *DlRan3B*, while the second half of these cDNA sequences were identical to the genomic sequence of *DlRan3A* (Fig. 2). In contrast, the first half of sequences of *DlRan3E*-*1* and *DlRan3G*-*1* were identical to the genomic sequence of *DlRan3A*, whereas the second half of these cDNA sequences was identical to the genomic sequence of *DlRan3B* (Fig. 2). Finally, the sequence of *DlRan3F*-*1* was inconsistent with either *DlRan3A* or *DlRan3B*. Our results suggest that these transcripts were encoded by different *DlRan* genes rather than representing alternative spliced products from the same gene, thereby implying the existence of multiple *Ran* genes in the longan genome.

All of the isolated *DlRan* transcript*s* encoded seven predicted polypeptides of 221 amino acid residues with similar calculated molecular masses and predicted pIs (Table 5). It is noteworthy that *DlRan3C*-*1*, *DlRan3C*-*2* and *DlRan3C*-*3*, which contain different ORFs, encoded the same protein. The modulation of protein expression via alteration of mRNA secondary structure has been demonstrated to involve the usage of synonymous codons (Nackley et al. 2006). We therefore used Mfold (Zuker 2003) to predict the secondary structures of the ORFs of these transcripts, which demonstrated that the Gibbs free energy for *DlRan3C*-*2* and *DlRan3C*-*3* was lower than that for *DlRan3C*-*1*.

**Table 5:** Calculated molecular mass and predicted pI of DlRan proteins

Protein name	Molecular weight (Da)	pI
DlRan3A	25,106.5	6.38
DlRan3B	25,150.6	6.75
DlRan3C	25,105.5	6.65
DlRan3D	25,159.6	6.65
DlRan3E	25,151.5	6.50
DlRAN3F	25,147.6	6.65
DlRAN3G	25,123.5	6.50

**Fig. 3:**
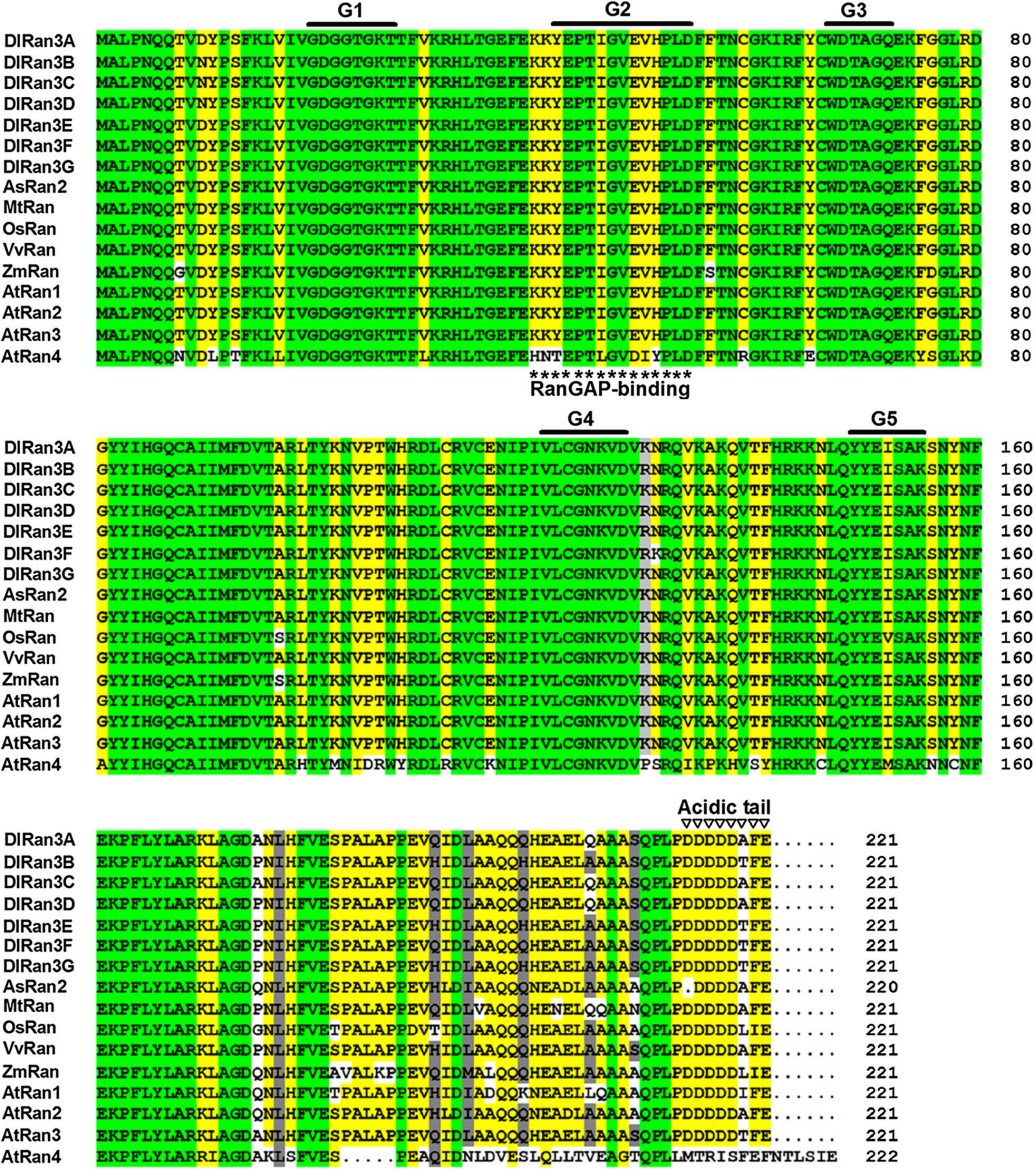
Multiple alignments of the deduced DlRan sequences with other Ran sequences. Sequences are from *A. thaliana* (AtRan1, NP_197501; AtRan2, NP_197502; AtRan3, NP_200330; AtRan4, NP_200319), *M. truncatula* (MtRan, ACJ83982), *Z. mays* (ZmRan, NP_001149221), *V. vinifera* (VvRan, XP_002284967), *A. cepa* (AsRan2, ABD17864) and *O. sativa* (OsRan, NP_001043550). Identical and similar amino acid residues among the aligned sequences are indicated by *green*, *yellow* and *grey shading*, respectively. Conserved GTP binding and hydrolysis domains (G1–G5) were indicated by *bold lines*. The effector-binding domain (RanGAP-binding) and the acidic C-terminal region (acidic tail) are indicated with *asterisks* and *triangles*, respectively

**Fig. 4:**
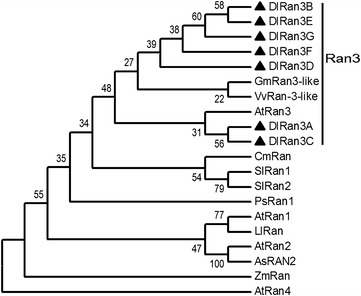
Phylogenetic relationships of Ran proteins from *D. longan* and selected plant species. Phylogenetic and evolutionary analyses were performed using the neighbor-joining method by MEGA5 software with 1000 bootstrap replicates. *A. thaliana* (AtRan1, NP_197501, AtRan2, NP_197502, AtRan3, NP_200330), *V. vinifera* (VvRan3-like, XP_002285018), *G. max* (GmRan3-like, XP_003526422), *Cucurbita maxima* (CmRan, AEK84227), *Solanum lycopersicum* (SlRan1, NP_001234016, SlRan2, NP_001234023), *Pisum sativum* (PsRan1, ABM73376), *Lepidium latifolium* (LlRan, AEK78856), *Allium sativum* (AsRan2, ABD17865), *Z. mays* (ZmRan, NP_001149221)

As shown in Additional file 5: Figure S5, alignment analysis revealed that the predicted DlRan proteins are highly identical to the identified peptides in our previous study (Fang et al. 2011). This result indicates that the predicted proteins were orthologs of the identified protein. DlRan members are highly similar to one another, differing by a total of only nine amino acids. Multiple sequence alignment indicated that the DlRan proteins share a significant degree of sequence identity with Ran proteins from *Arabidopsis thaliana*, *Medicago truncatula*, *Zea mays*, *Vitis vinifera*, *Allium cepa* and *Oryza sativa* (Fig. 3). The characteristic domains of the Ran proteins that are known to be involved in GTP-binding and hydrolysis, as well as the acidic C-terminal domain and the effector-binding domain, were detected in the deduced DlRan proteins (Fig. 3). As shown in Fig. 3, the conserved sequences of these motifs were nearly identical between DlRan proteins and Ran proteins from other plant species, except for AtRan4, which has distinct functions in Arabidopsis (Vernoud et al. 2003). In the neighbor-joining phylogenetic tree based on the DlRan proteins and Ran proteins from multiple plant species, the DlRan proteins, AtRan3 and Ran3-like proteins from *Glycine max* and *V. vinifera* were clustered into one group (Fig. 4). These results suggest that the DlRan proteins are Ran3 homologs.

### Expression analysis of *DlRan* genes during SE in longan

**Fig. 5:**
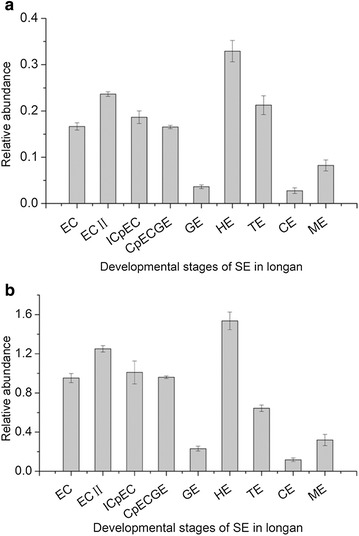
Relative expression levels of *DlRan* genes during longan somatic embryogenesis determined by qRT-PCR. Expression level was normalized to *Fe-SOD* and *EF*-*1a*. Data are mean ± SE (n = 3). **a** Expression level of N type *DlRan* transcripts (*DlRan3B*-*1*–*DlRan3B*-*9*, *DlRanC*-*1*–*DlRan3C*-*3*, *DlRanD*-*1*and *DlRanD*-*2*). **b** Expression level of D type *DlRan* transcripts (*DlRan3A*-*1*–*DlRan3A*-*14*, *DlRanE*-*1*, *DlRanF*-*1* and *DlRanG*-*1*). *EC* friable-embryogenic callus, *EC II* embryogenic callus II, *ICpEC* incomplete compact pro-embryogenic cultures, *CpECGE* compact proembryogenic cultures, *GE* globular embryos, *HE* heart-shaped embryos, *TE* torpedo-shaped embryos, *CE* cotyledonary embryos, *ME* mature embryos. Morphology of these embryogenic cultures has been described in previous studies (Lai et al. 2012; Lai and Lin 2013)

We used qRT-PCR to detect abundances of *DlRan* transcripts at different developmental stages of longan SE. As indicated in Fig. 5, the expression profiles of two types of *DlRan* genes during longan SE were very similar. High levels of *DlRan* transcripts were detected in early embryogenic cultures and heart- and torpedo-shaped embryos. The highest levels were found in heart-shaped embryos, while the lowest were detected in globular, cotyledonary and mature embryos.

### The effect of 2,4-D on expression of *DlRan* genes in longan embryogenic calli

**Fig. 6:**
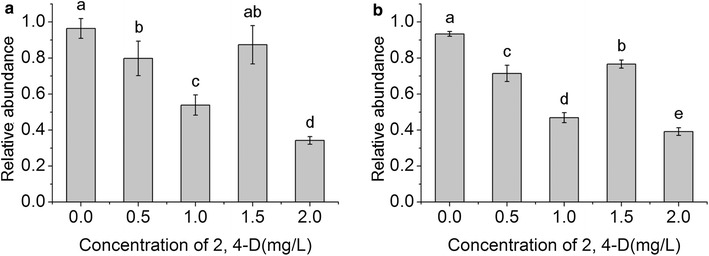
Expression of *DlRan* genes under 2, 4-D treatment. Embryogenic calli were treated with M0 supplemented with 0.5, 1.5 and 2.0 mg/l of 2,4-D and 2,4-D free medium, respectively. RNA was extracted from embryogenic calli and analyzed by realtime PCR to determine the relative abundance of *DlRan* genes. **a** Abundance of N type *DlRan* transcripts, **b** abundance of D type *DlRan* transcripts. Abundance was normalized to *Fe-SOD* and *EF*-*1a*. Significance was tested by one-way ANOVA using SPSS 13.0. *Different letters above the bars* indicate significant differences according to the least significant difference test at 5 % level. Data are mean ± SE (n = 3)

2,4-D is a growth regulator commonly used in the induction of somatic embryos. However, high concentrations inhibit development of somatic embryos in longan and other plants (Aiqing et al. 2011; Lai et al. 2000). Furthermore, application of 2,4-D in various concentrations is able to synchronize SE in longan (Chen and Lai 2002). Wang et al. (2006) have demonstrated that *Ran* is involved in auxin signaling. 1 mg 2,4-D/l is necessary to maintain longan calli at embryogenic state (Lai et al. 2000). To investigate the effect of 2,4-D on the expression of *DlRan* genes, embryogenic calli cultured on M0 medium supplemented with 1 mg 2,4-D/l were transferred to M0 medium supplemented with different concentrations of 2,4-D. As indicated in Fig. 6, reducing the concentration of 2,4-D gradually increased the abundance of *DlRan* gene transcripts. Increasing the concentration of 2,4-D to 1.5 mg/l also enhanced the accumulation of *DlRan* genes transcripts. In contrast, application of 2.0 mg 2,4-D/l reduced the abundance of *DlRan* transcripts to levels lower than initial values.

## Discussion

### Characterization of an expanded *Ran* gene family in longan

The *Ran* gene family comprises a small number of genes found in different organisms, namely one member in humans and *Schizosaccharomyces pombe* and four in Arabidopsis (Ma 2007; Takai et al. 2001). In this study, 30 *DlRan* cDNAs were cloned from torpedo-shaped embryos in longan. Alignments between *DlRan* cDNA sequences and genomic DNA sequences suggested the existence of more *Ran* genes in the longan genome. Phylogenetic analysis revealed that seven deduced DlRan proteins are closely related to Ran3 from other species. Our results suggest that the longan *Ran* gene family is expanded compared with Arabidopsis (Ma 2007). The estimated size of the longan genome is 444 Mb (VanBuren et al. 2011), about threefold larger than that of Arabidopsis. Nevertheless, the exact number of *Ran* genes in longan cannot be determined until whole genome sequencing is completed. Sequence features of the longan *Ran* gene family that may be unique to this species and cannot be determined until all *Ran* genes have been isolated from the longan genome.

### Regulation of *DlRan* gene expression

In the present study, *DlRan* genes were significantly upregulated at the heart-shaped embryo stage. At the torpedo-shaped embryo stage, *DlRan* genes were downregulated whereas the Ran protein was rapidly upregulated. Our results indicate that the expression patterns of *DlRan* genes were different from that of the Ran protein identified in our previous study (Fang et al. 2011; Lai et al. 2012). Discordance between protein and mRNA expression is a common phenomenon in eukaryotic cells (Skrzycki et al. 2010; Wang et al. 2010). We speculate that unidentified post-transcriptional mechanisms participate in regulation of *DlRan* gene expression.

We found that changes in synonymous codon usage gave rise to mRNA secondary structure alterations among *DlRan3C*-*1*, *DlRan3C*-*2* and *DlRan3C*-*3*. Although synonymous mutations have no effect on the resulting protein sequence, the selection of synonymous codons affects the modulation of gene expression and cellular functions (Plotkin and Kudla 2011). The differential usage of synonymous codons among these transcripts may be functional, but further tests are required to confirm this hypothesis.

### Potential functions of *DlRan* genes during SE in longan

The involvement of Ran in longan SE has been demonstrated previously (Fang et al. 2011). Our results indicated that reduction of 2,4-D concentration in the medium, which promotes initiation of somatic embryo development, enhanced *DlRan* gene expression. This result further supports the involvement of *DlRan* in longan SE. Plant Ran is involved in cell proliferation (Lü et al. 2011; Wang et al. 2006). The sequence alignment in the present study indicates that DlRan proteins are highly conserved with respect to Ran proteins from other plants, suggesting similar functionality. Our expression analysis showed that *DlRan* gene transcripts are more abundant during SE stages associated with active cell division. The high expression of *DlRan* genes observed at heart- and torpedo-shaped stages may be related to the cell proliferation that gives rise to the cotyledons and radicle. We believe that DlRan proteins may regulate mitotic progress in a manner similar to their homologs in other plants.

2,4-D was shown to alter *Ran* expression when applied at different concentrations. Auxin plays pivotal roles in SE. 2,4-D, the most commonly used synthetic auxin for induction of SE (Karami and Saidi 2010), affects the indole acetic acid (IAA) synthetic pathway and promotes IAA accumulation (Michalczuk et al. 1992a, b). Ectopic postembryonic expression of *LEC2* has been shown to induce somatic embryo formation (Stone et al. 2001). LEC2 has been proposed to induce SE by promoting auxin activity, and 2,4-D exerts effects similar to those of ectopic *LEC2* expression (Stone et al. 2008). Su et al. (2009) have suggested that exogenous auxin levels play an important role in determining expression patterns of *WUS*, a correct expression of which is essential for somatic embryo induction. 2,4-D can induce SE, but also inhibits somatic embryo development (Aiqing et al. 2011). Pan et al. (2010) found that treatment with high concentrations of 2,4-D changed the proteome of *Valencia* embryogenic callus. Although the mechanisms involved in induction of SE by 2,4-D and the inhibitory effect of this auxin on somatic embryo development remain to be uncovered, 2,4-D functions by altering gene expression in plant cells through signal transduction. Ran is a vital regulator of nucleocytoplasmic trafficking in plants (Meier and Somers 2011; Merkle 2011). Numerous studies have detailed the involvement of Ran in plant responses to hormonal and environmental signaling (Ferreira et al. 2006; Jiang et al. 2007; Kriegs et al. 2006; Lee et al. 2008; Mahong et al. 2012; Wang et al. 2006; Xu and Huang 2010; Yoshimura et al. 2008). *Ran* is involved in auxin signaling (Wang et al. 2006) and it is unsurprising to find that *Ran* expression is influenced by 2,4-D. 1 mg 2,4-D/l is necessary to maintain longan calli at embryogenic state, remove or reduce the concentration of 2,4-D initiates the development of somatic embryos. Nucleocytoplasmic transport and cell division are essential during the formation of somatic embryos. It is reasonable that the expression of *Ran* was enhanced by reducing the concentration of 2,4-D. Properly increasing the concentration of 2,4-D promote the proliferation of longan calli and improve the expression of *Ran*. However, 2 mg 2,4-D/l inhibit the growth of longan calli and cause browning, which can explain the repression effect of 2 mg 2,4-D/l on *Ran* level. Our results further support the involvement of *Ran* in auxin signal transduction. Zang et al. (2010) have suggested that *Ran* participates in abiotic response signaling by modulating the nuclear transportation of proteins and RNA. Taking the results of these studies and ours into consideration, we speculate that *DlRan* may participate in 2,4-D-induced SE by transmitting 2,4-D signals and may regulate the expression of embryogenesis-related genes by controlling nuclear trafficking.

In this study, 30 cDNA and two genomic DNA sequences of *DlRan* genes were isolated. We also revealed the expression profiles of *DlRan* genes during SE and under exogenous 2,4-D treatment. Our results suggest the importance of *DlRan* genes in longan embryo development. Future research should focus on the elucidation of mechanisms involved in regulation of *DlRan* gene expression and the functions of different *DlRan* genes during SE in longan.

## Additional files

10.1186/s40064-016-1887-0 Schematic of RACE primer locations. Arrows indicate the locations of RACE primers.
10.1186/s40064-016-1887-0 Location of binding sites for qRT-PCR primers.
10.1186/s40064-016-1887-0 Alignment of 3′ ends of *DlRan* cDNAs.
10.1186/s40064-016-1887-0 Alignment of 5′ ends of *DlRan* cDNAs.
10.1186/s40064-016-1887-0 Multiple sequence alignment of DlRan proteins and previously identified Ran peptides.
