# Supplementary material for: Molecular cloning, structural and expression profiling of DlRan genes during somatic embryogenesis in Dimocarpus longan Lour
Source: Springerplus. 2016 Feb 25;5:181. doi: 10.1186/s40064-016-1887-0 (PMC4766155; doi:10.1186/s40064-016-1887-0)
Supplement: Supplementary file 1 — 10.1186/s40064-016-1887-0 Schematic of RACE primer locations. Arrows indicate the locations of RACE primers. [file 40064_2016_1887_MOESM1_ESM.doc]

**
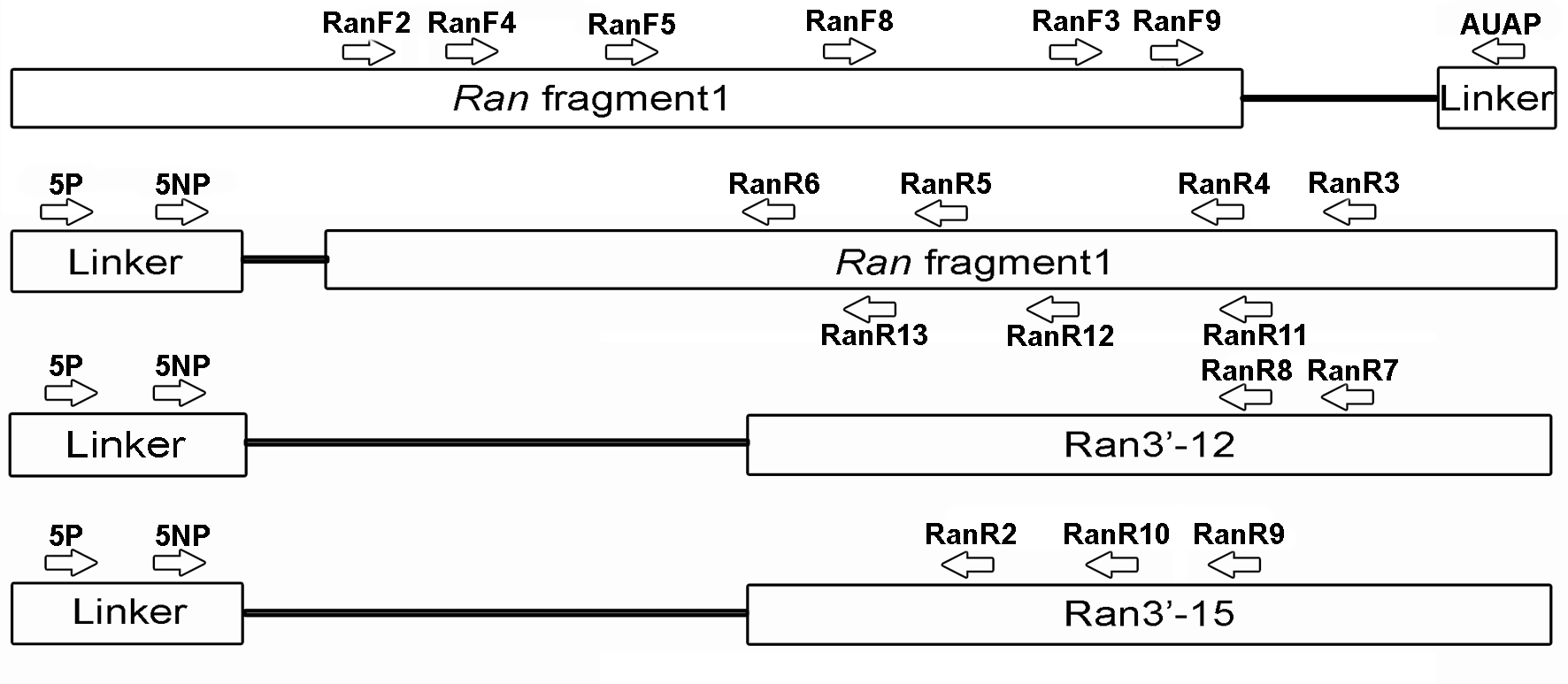
**

**Figure S1. Schematics demonstration of the RACE primer locations.** Arrows indicate the locations of RACE primers.
