# Supplementary material for: Molecular cloning, structural and expression profiling of DlRan genes during somatic embryogenesis in Dimocarpus longan Lour
Source: Springerplus. 2016 Feb 25;5:181. doi: 10.1186/s40064-016-1887-0 (PMC4766155; doi:10.1186/s40064-016-1887-0)
Supplement: Supplementary file 2 — 10.1186/s40064-016-1887-0 Location of binding sites for qRT-PCR primers. [file 40064_2016_1887_MOESM2_ESM.doc]

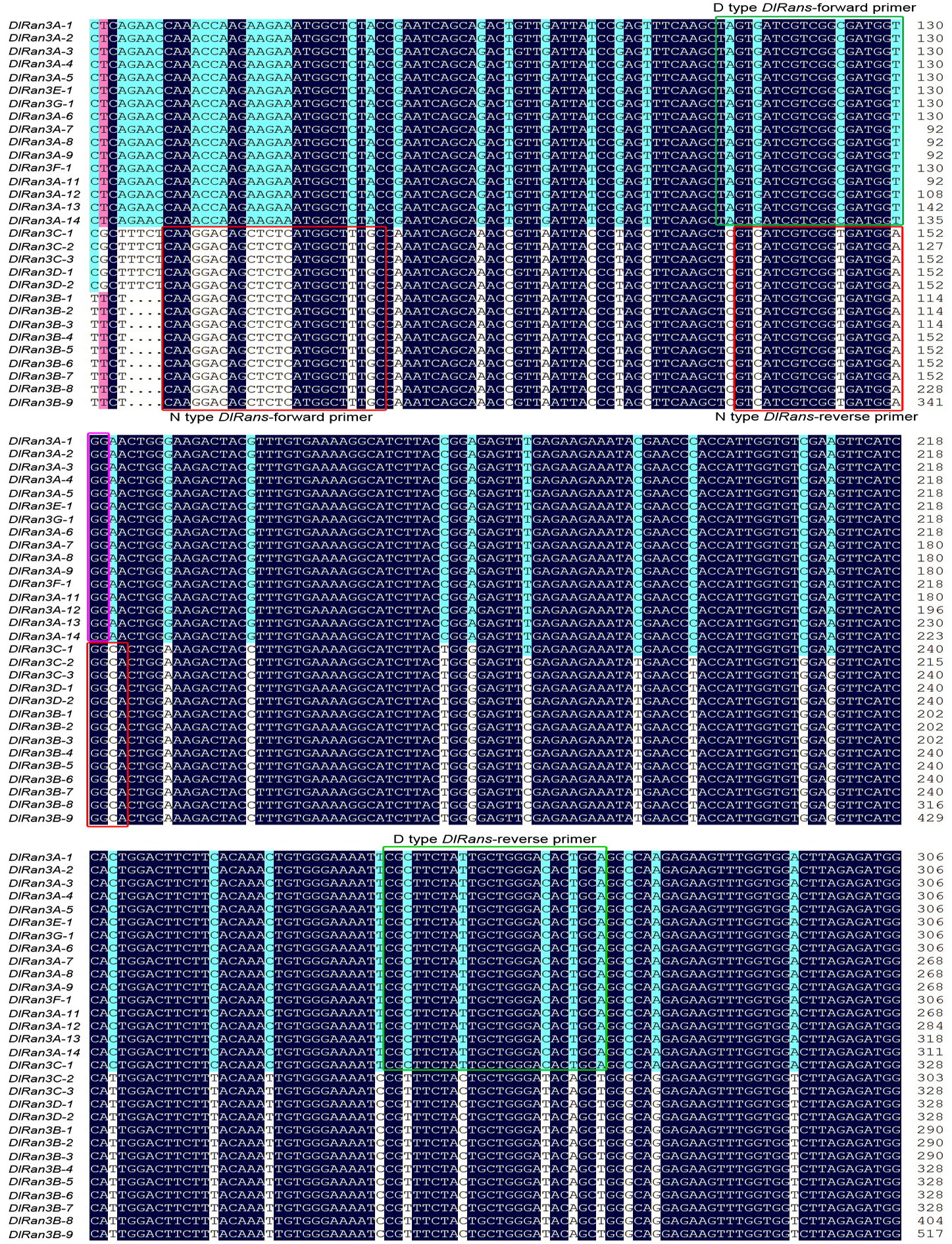


**Figure S2. Location of binding sites for qRT-PCR primers.** Binding sites for primer of D type *DlRan* transcripts are indicated by green boxes and that of N type *DlRan* transcripts are indicated by red boxes.
