# Supplementary material for: Molecular cloning, structural and expression profiling of DlRan genes during somatic embryogenesis in Dimocarpus longan Lour
Source: Springerplus. 2016 Feb 25;5:181. doi: 10.1186/s40064-016-1887-0 (PMC4766155; doi:10.1186/s40064-016-1887-0)
Supplement: Supplementary file 3 — 10.1186/s40064-016-1887-0 Alignment of 3′ ends of DlRan cDNAs. [file 40064_2016_1887_MOESM3_ESM.doc]

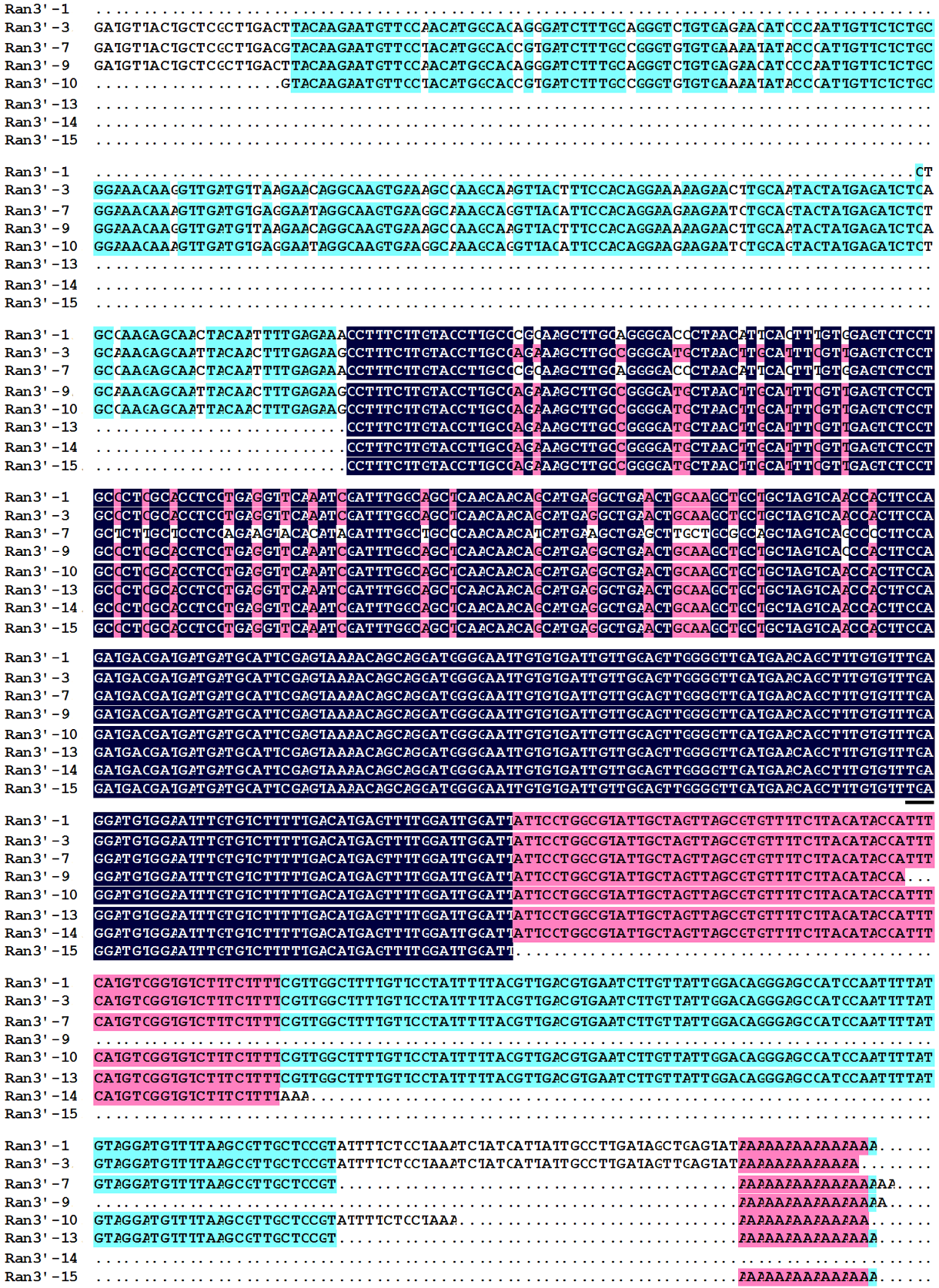


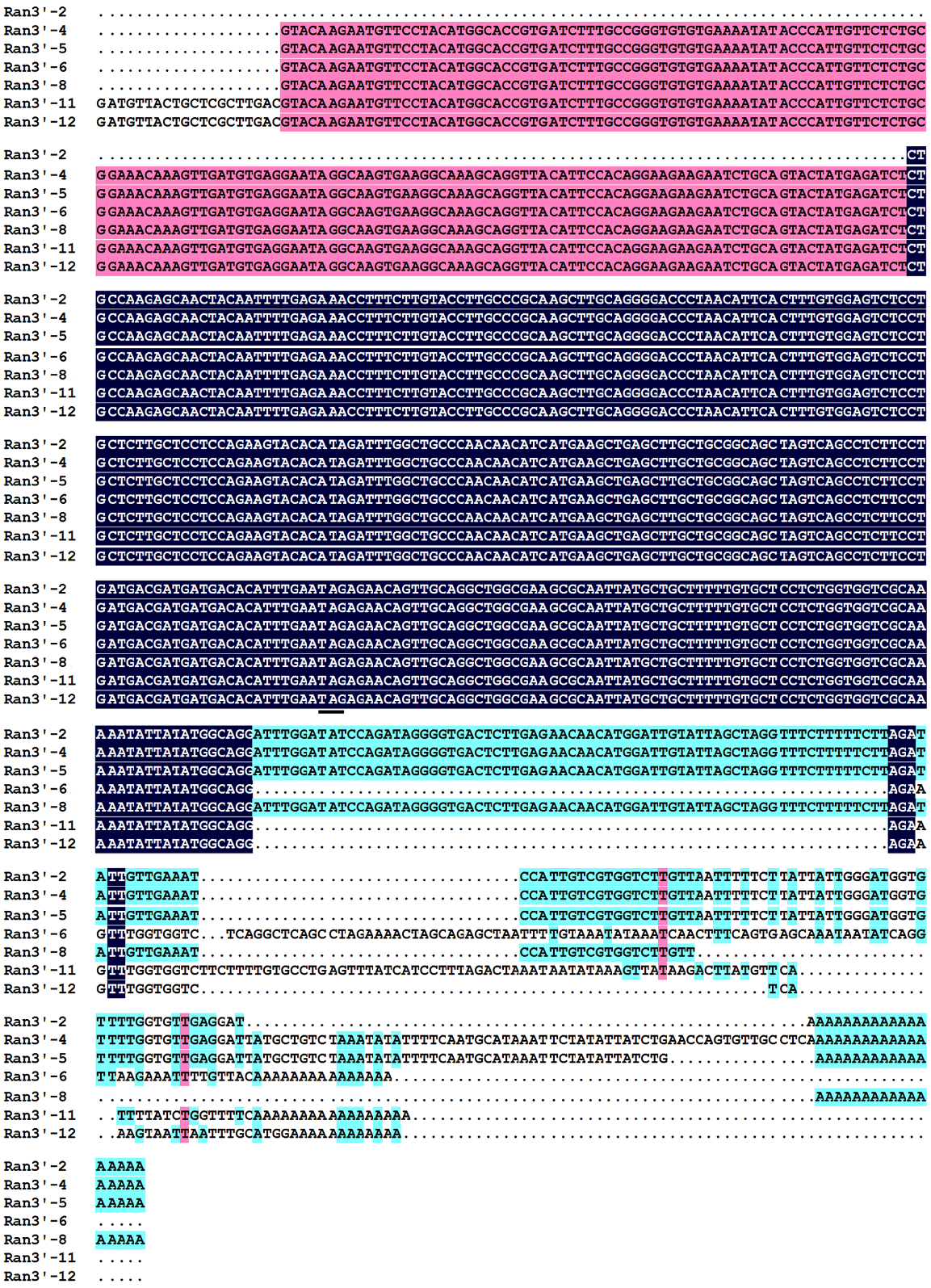


**Figure S3. Alignment of 3' ends of DlRan cDNAs** Termination codons are underlined. Identical nucleotides among the aligned sequences are indicated by black, blue and pink shading, respectively.
