# Supplementary figures and images for: Molecular cloning, structural and expression profiling of DlRan genes during somatic embryogenesis in Dimocarpus longan Lour
Source: Springerplus. 2016 Feb 25;5:181. doi: 10.1186/s40064-016-1887-0 (PMC4766155; doi:10.1186/s40064-016-1887-0)

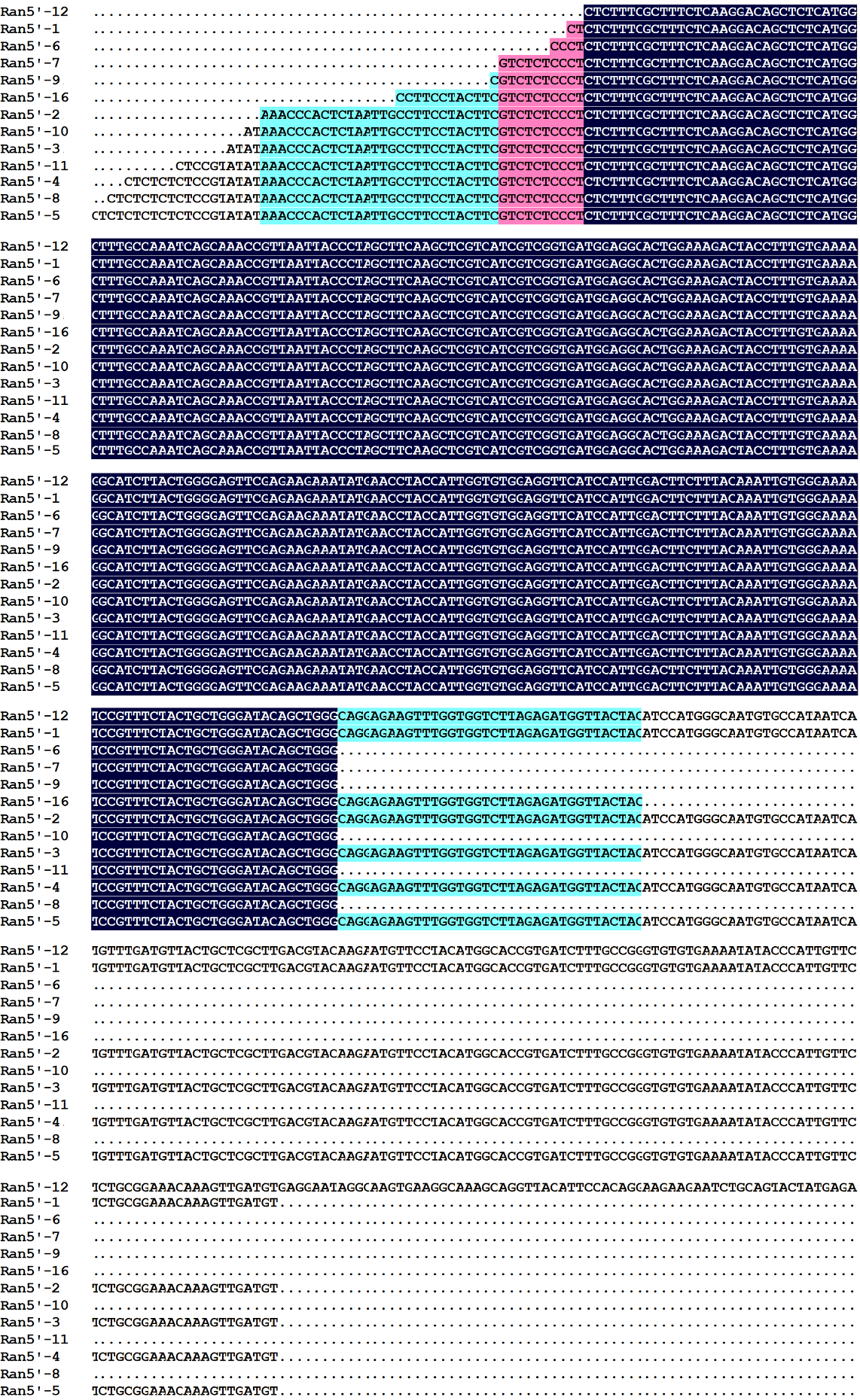


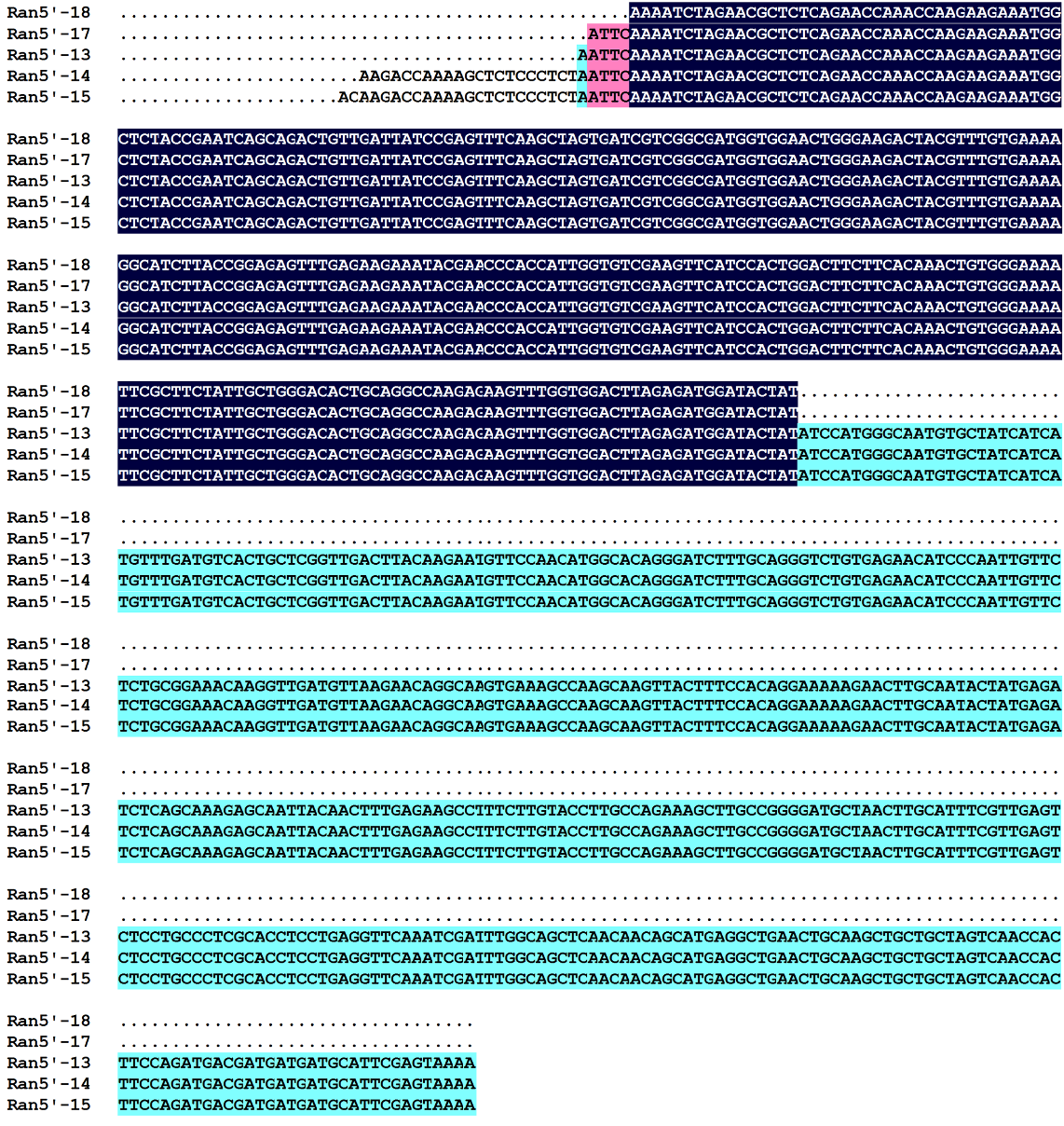


**Figure S4. Alignment of 5'ends of DlRanAN cDNAs.** Start codons are underlined.

Supplement: Supplementary file 4 — 10.1186/s40064-016-1887-0 Alignment of 5′ ends of DlRan cDNAs. [file 40064_2016_1887_MOESM4_ESM.doc]
