# Supplementary material for: Molecular cloning, structural and expression profiling of DlRan genes during somatic embryogenesis in Dimocarpus longan Lour
Source: Springerplus. 2016 Feb 25;5:181. doi: 10.1186/s40064-016-1887-0 (PMC4766155; doi:10.1186/s40064-016-1887-0)
Supplement: Supplementary file 5 — 10.1186/s40064-016-1887-0 Multiple sequence alignment of DlRan proteins and previously identified Ran peptides. [file 40064_2016_1887_MOESM5_ESM.doc]

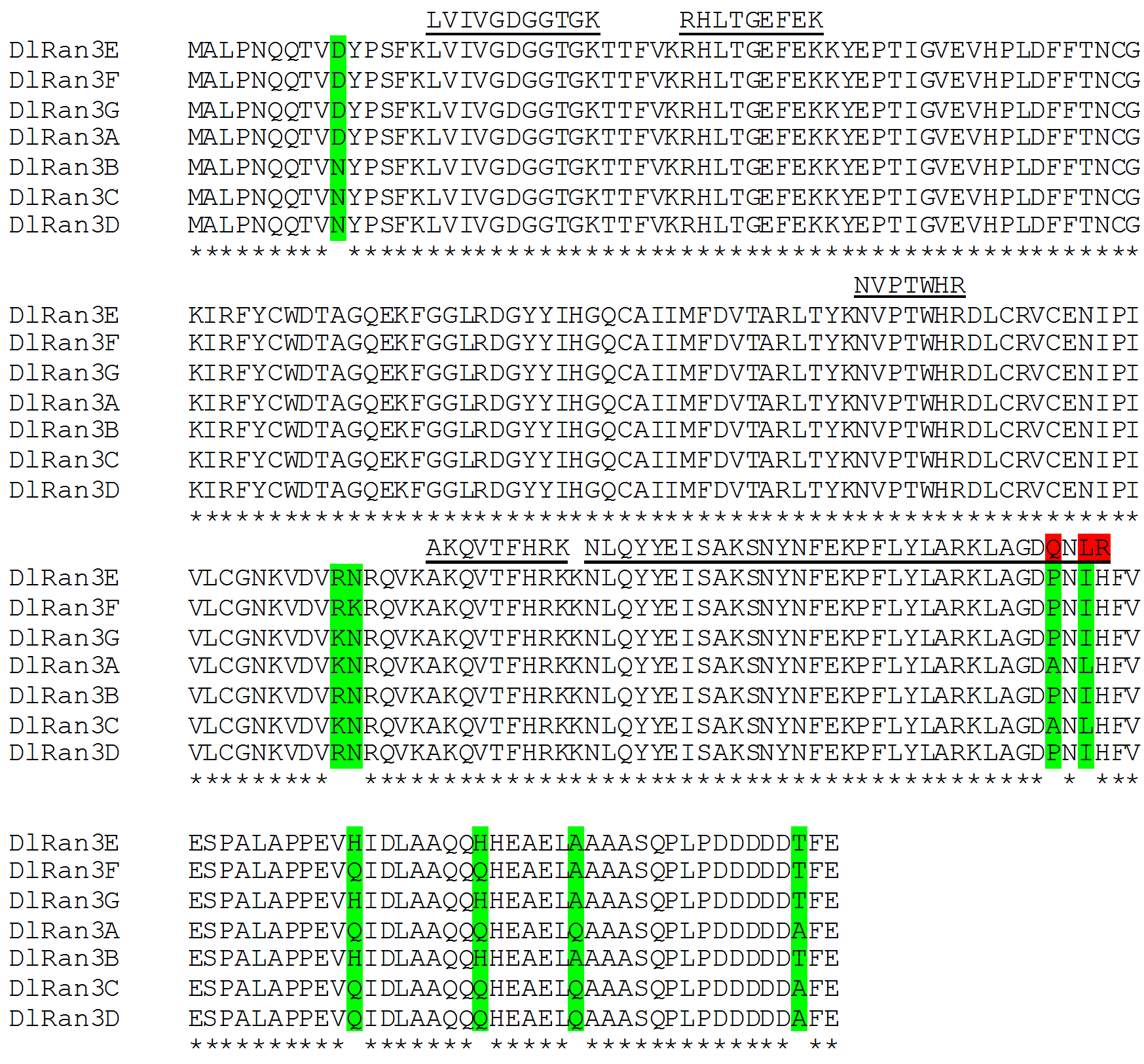


**Figure S5. Multiple sequence alignment of DlRan proteins and previously identified Ran peptides.** The identified peptides are are underlined, identical amino acids are marked with asterisks, different amino acids among DlRan proteins are green shaded, different amino acids between identified peptides and DlRan proteins are indicated by red shading.
